# Hyperkalemic cardioplegia for adult and pediatric surgery: end of an era?

**DOI:** 10.3389/fphys.2013.00228

**Published:** 2013-08-28

**Authors:** Geoffrey P. Dobson, Giuseppe Faggian, Francesco Onorati, Jakob Vinten-Johansen

**Affiliations:** ^1^Department of Physiology and Pharmacology, Heart and Trauma Research Laboratory, James Cook UniversityTownsville, QLD, Australia; ^2^Division of Cardiac Surgery, University of Verona Medical SchoolVerona, Italy; ^3^Cardiothoracic Research Laboratory of Emory University Hospital Midtown, Carlyle Fraser Heart CenterAtlanta, GA, USA

**Keywords:** cardioplegia, ischemia, cardiac surgery, heart, potassium, hyperkalemia, endothelium, history

## Abstract

Despite surgical proficiency and innovation driving low mortality rates in cardiac surgery, the disease severity, comorbidity rate, and operative procedural difficulty have increased. Today's cardiac surgery patient is older, has a “sicker” heart and often presents with multiple comorbidities; a scenario that was relatively rare 20 years ago. The global challenge has been to find new ways to make surgery safer for the patient and more predictable for the surgeon. A confounding factor that may influence clinical outcome is high K^+^ cardioplegia. For over 40 years, potassium depolarization has been linked to transmembrane ionic imbalances, arrhythmias and conduction disturbances, vasoconstriction, coronary spasm, contractile stunning, and low output syndrome. Other than inducing rapid electrochemical arrest, high K^+^ cardioplegia offers little or no *inherent* protection to adult or pediatric patients. This review provides a brief history of high K^+^ cardioplegia, five areas of increasing concern with prolonged membrane K^+^ depolarization, and the basic science and clinical data underpinning a new normokalemic, “polarizing” cardioplegia comprising adenosine and lidocaine (AL) with magnesium (Mg^2+^) (ALM™). We argue that improved cardioprotection, better outcomes, faster recoveries and lower healthcare costs are achievable and, despite the early predictions from the stent industry and cardiology, the “cath lab” may not be the place where the new wave of high-risk morbid patients are best served.

## Introduction

### In-hospital mortality rates: only the tip of the iceberg

“Declare the past, diagnose the present, foretell the future; practice these acts. As to diseases, make a habit of two things—to help, or at least to do no harm.”

Attributed to Hippocrates (Epidemics, Bk.1, Sect. XI) (Markel, 2004)

**Figure 1:**
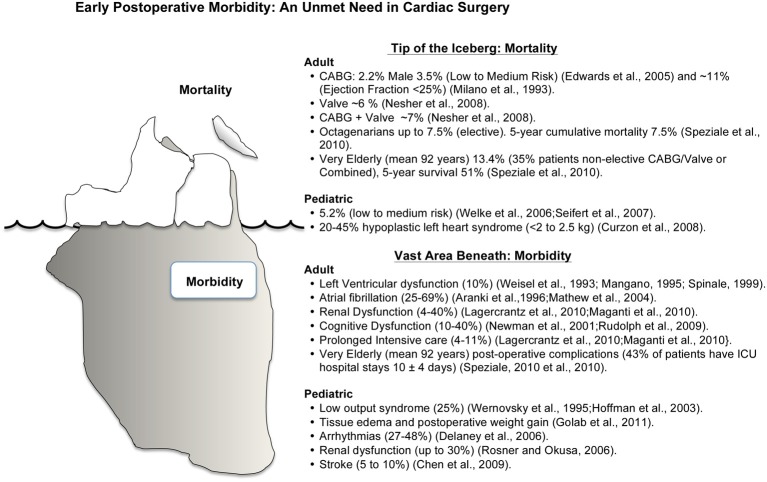
**Schematic representation of mortality and morbidity in cardiac surgery.** Mortality and morbidity have many interacting and confounding factors with ischemia-reperfusion injury playing a significant role in the underlying etiologies. Improving early post-operative morbidity is an unmet need in cardiac surgery (see text for Discussion).

Coronary artery disease is responsible for approximately one-third of the world's population deaths over 35 years of age (Mangiacapra et al., 2011). Globally there are over 800,000 coronary artery bypass graft (CABG) surgery or valvular operations each year, with around 1000 operations each day in the US (Goldman et al., 2004; Thom et al., 2006; Dobson, 2010). In-hospital/30-day mortality rates are around 1% for CABG, 5–6% for valve, and 7% for combined CABG and valve surgery (Nesher et al., 2008; Hannan et al., 2013) (Figure 1). These mortality rates are not equivalent across hospitals (Goodney et al., 2003), age (Vicchio et al., 2012), gender (Edwards et al., 2005), ethnicity (Becker and Rahimi, 2006; Song et al., 2008) or countries (Menasché, 2000). Octogenarians have a 3-fold increased risk of death (~7.5%) compared with younger adults (Speziale et al., 2011), and 90 year old, non-elective, patients have a mortality ranging between 7 and 18% with a five year survival of 51% (Speziale et al., 2010) (Milano et al., 1993). Adult females have up to 1.6 times higher in-hospital mortality rates and higher morbidity than their male counterparts (Edwards et al., 1998, 2005; Vaccarino et al., 2002; Koch et al., 2003; Azakie and Russell, 2004). The increased risks in woman are believed to involve greater perioperative susceptibility to ischemia-reperfusion (IR) injury, different myocyte-endothelial physiology, different hormonal status, higher incidence of diabetes, hypertension and renal disease, and the effect of age and ethnicity, with worse outcomes among Afro-Americans (Edwards et al., 2005; Seifert et al., 2007; Martin et al., 2012).

Pediatric patients also have a unique set of surgical and post-operative challenges. Complex congenital corrective operations have an in-hospital mortality rate of ~5.0% and, as in adults, the female gender appears to be an independent risk factor for higher mortality and adverse events (1.3 times higher in-hospital mortality) (Welke et al., 2006; Seifert et al., 2007). In high-risk, low weight, pediatric patients (weighing < 2–2.5 kg) hypoplastic left heart syndrome has an in-hospital mortality of 20–45% (Curzon et al., 2008) (Figure 1).

### Post-operative morbidity: the vast area beneath the surface

Despite surgical proficiency and medical innovation driving low mortality rates, the disease severity, operative procedural difficulty, and comorbidity rate have all increased in recent years (Figure 1). About 10% of CABG surgery patients will have significant left ventricular dysfunction lasting several hours, days, or weeks (Weisel, 1993; Mangano, 1995; Spinale, 1999). Twenty five to 40% of patients will have post-operative atrial fibrillation (Aranki et al., 1996; Mathew et al., 2004), and in those patients undergoing *combined* CABG and valvular surgery the incidence may increase to 69% (Scherr et al., 2006). In the USA alone, atrial fibrillation costs healthcare providers over 1.5 billion dollars per year (Steinberg, 2004), and this cost likely doubles on a global scale. In addition, depending upon the type of surgery, 4–40% of patients will have some form of renal dysfunction (Lagercrantz et al., 2010; Maganti et al., 2010; Shaw, 2012), and 10–40% of adult patients will experience transient cognitive dysfunction or delirium, which can last for up to 5 years (Newman et al., 2001; Rudolph et al., 2009), and 2–13% patients will have a stroke (Kellermann and Jungwirth, 2010). Perioperative bleeding is another major complication of cardiac surgery and excessive bleeding occurs around 20% of patients, and 5–7% will lose in excess of 2 L within the first 24 h postoperatively (Raja, 2005; Yavari and Becker, 2009). It has been estimated that about 50% of blood loss is due to identifiable surgical bleeding, and the other 50% is due to a complex hypocoagulopathy associated with surgical trauma and cardiopulmonary bypass (Raja, 2005; Yavari and Becker, 2009).

Among the very elderly, over 40% will experience significant post-operative complications requiring extended ICU stays often involving many weeks (Speziale et al., 2010). Similarly, in pediatric patients undergoing complex congenital corrective operations, many will have acute post-operative complications such as tissue edema with postoperative weight gain (Golab et al., 2011), systemic coagulation disorders (Hayash et al., 2011), surgical complications and low output syndrome (up to 25%) (Wernovsky et al., 1995; Hoffman et al., 2003), arrhythmias (27–48%) (Delaney et al., 2006), renal dysfunction (up to 30%) (Rosner and Okusa, 2006), and cerebral dysfunction and stroke (5–10%) (Chen et al., 2009).

### Role of ischemia-reperfusion (IR) injury

Prior to the 1990s, there was considerable skepticism among cardiologists and some cardiac surgeons regarding the clinical authenticity of myocardial ischemia-reperfusion injury. However, over the last two decades, IR injury has been recognized as a significant contributor to mortality and morbidity in cardiac surgery patients (Vaage and Valen, 1993; Vinten-Johansen and Nakanishi, 1993; Anselmi et al., 2004; Beyersdorf, 2009), with females being more susceptible than males (Abramov et al., 2000; Butterworth et al., 2000; McCully et al., 2006; Doughtie et al., 2010). Consequences of IR injury are exacerbated by physiological responses to cardiopulmonary bypass and surgical trauma caused by release of pro-inflammatory mediators. Hence, as opposed to IR injury found in percutaneous coronary interventions, cardiac surgery represents a “three-hit” model of potential injury to the heart: ischemia-reperfusion, cardiopulmonary bypass and surgical trauma (Weman et al., 2000). It may be more accurate to redefine reperfusion injury as “postcardioplegic” injury (Vinten-Johansen and Nakanishi, 1993). Postcardioplegic injury is particularly prevalent during and following the surgical trauma associated with adult and pediatric cardiac surgery (Anselmi et al., 2004; Madhok et al., 2006; Welke et al., 2006; Seifert et al., 2007; Butler et al., 2009; Gessler et al., 2009).

Post-cardioplegic injury is dependent on pre-operative and/or intra-operative myocardial ischemia; *without ischemia there is no reperfusion injury*. Injury is classified as reversible or irreversible. Reversible injury includes arrhythmias, edema, vascular dysfunction, and contractile stunning expressed as low output syndrome requiring inotropic or mechanical support to maintain acceptable hemodynamics (Vinten-Johansen and Nakanishi, 1993; Rudd and Dobson, 2011a). Irreversible reperfusion injury includes necrosis and apoptosis (Anselmi et al., 2004, 2009). Tissue edema and microvascular dysfunction can transition from reversible to irreversible (i.e., no-reflow) in response to severe or prolonged ischemia, and can contribute to ultimate necrosis of myocardium. Necrosis involves disruption or disintegration of the cell membrane, and the release of cell contents and large proteins that are used as biomarkers indicative of morphological injury, e.g., creatine kinase (CK, CK-MB) or cardiac troponins (T or I subunits). Peri-operative cell death is substantiated by the release of these biomarkers into the plasma (Costa et al., 2001; Lehrke et al., 2004). Numerous studies have shown that these elevations in perioperative CK-MB or cTn (within 24–48 h) are correlated with both short-term and long-term risk of mortality (Klatte et al., 2001; Lehrke et al., 2004; Domanski et al., 2011). This increase in biomarkers after surgery has been ascribed in part to suboptimal intra-operative myocardial protection. In contrast to necrosis, apoptosis, or programmed cell death, is non-explosive, non-inflammatory cell death initiated over a longer period of time than the more rapid onset of necrosis.

#### Mechanisms of IR injury

Reperfusion injury is a process, and not an endpoint. The process of Ischemia-reperfusion is associated with cell K^+^ efflux, membrane depolarization, anaerobic lactate production and ATP decline, intracellular H^+^, Na^+^, and Ca^2+^ loading, ionic imbalances, cell swelling, free radical and oxidant production, mitochondrial pore opening and dysfunction, apoptosis and necrosis (Piper et al., 2003, 2004; Cannon, 2005; Vinten-Johansen et al., 2007b). Ischemia-reperfusion is also responsible for endothelial activation leading to microvascular dysfunction and deterioration of coronary flow reserve (Ferguson et al., 1996; Feng et al., 2008), vasoconstriction and spasm (Ruel et al., 2004), myocardial contractile dysfunction (Weisel, 1993; Allen, 2004), reperfusion arrhythmias (Anselmi et al., 2004, 2009), activation of the inflammation cascade (cytokines, chemokines, complement activation, neutrophil activation) (Levy and Tanaka, 2003; Merchant et al., 2008; Suleiman et al., 2008; Anselmi et al., 2009) and coagulation imbalances (Raivio et al., 2009; Yavari and Becker, 2009). Reperfusion following cardiac surgery has shown to be associated with a significant thrombin generation followed by activation of protein C (Raivio et al., 2009).

#### Potassium in cardioplegia and IR injury

Another factor that may exacerbate IR injury is high potassium in cardioplegia. Each year, over four metric tons of depolarizing potassium (K^+^) are perfused through the coronary arteries of cardiac patients worldwide, and about two metric tons in USA patients (Dobson, 2010). After presenting a brief history of K^+^ cardioplegia, we will discuss how prolonged K^+^-dependent cellular depolarization can create a hostile, unnatural pro-ischemic environment for the heart, which may contribute to damage and post-operative morbidity in the cardiac surgery patient. Next, we will discuss alternatives to high K^+^ cardioplegia including a new polarizing cardioplegia and reanimation solution comprising adenosine and lidocaine with Mg^2+^ (ALM™) (Dobson, 2004) that may address the unmet needs of the new high-risk morbid patient.

### Brief history of cardioplegia: from ringer to cardiac surgery

If too little potassium is present, the contractions become broader, and there results in fusion of the beats. If too much potassium is present … then the contraction of the ventricle is imperfect, and by increasing the quantity of potassium salt the beat becomes weaker and weaker till it stops.

Sidney Ringer (1883) From Moore (Moore, 1911) pvii

**Figure 2:**
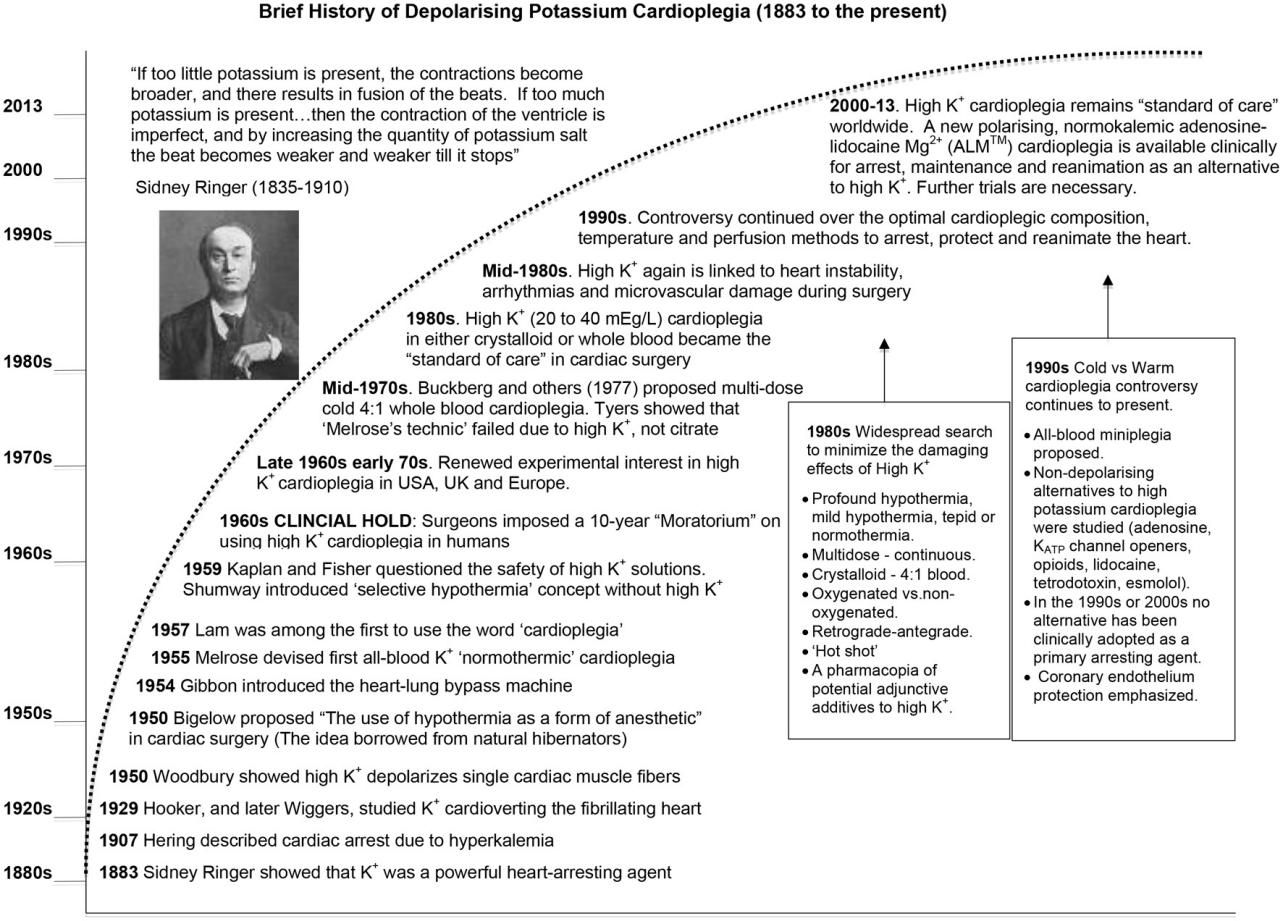
**A brief history of the development of potassium cardioplegia from basic science to cardiac surgery (See text for details)**.

The term cardioplegia (*cardio*, heart and *plegia*, paralysis) was first introduced by Lam in 1957 (Lam et al., 1957), yet the method of arrest has its roots in the early experiments of British physiologist Sidney Ringer using the frog heart (Figure 2). In 1883 Ringer reported that potassium chloride was a powerful arresting agent (Ringer, 1883), a serendipitous discovery like many in science and medicine (Dobson, 2005). Curiously, Ringer's laboratory had a malfunctioning water distiller, and his technician prepared the heart solutions using tap water supplied by London's New River Water Company (Moore, 1911). The technician found that hearts perfused with this tap water-based solution contracted rhythmically and forcefully for a number of hours. After identifying the anomalies between tap water that was high in calcium and magnesium salts, and distilled water, Ringer had the experiments repeated using distilled water, and the hearts quickly failed. To maintain contraction it was necessary to add Ca^2+^ and other salts to the crystalloid medium (Moore, 1911). By accident Ringer and his team next discovered the importance of potassium in “stilling” the heart in diastole and calcium in “stimulating” the heart in systole (see quote above).

A few decades later, Hering (1907) reported the association between high serum potassium and emergent “fibrillary” contractions, which led to cardiac arrest (Hering, 1953). In 1929, Hooker extended these observations and showed in a fibrillating dog heart that injecting 1 ml of 2.5% KCl (336 mM) into the coronary arteries rapidly stopped the heart, and after washout the heart returned to sinus rhythm (Hooker, 1929). Wiggers showed that an intravenous administration of calcium after KCl “cardioversion,” led to a more stable recovery (Wiggers, 1950). In 1954, Montgomery used a similar KCl approach in hypothermic human patients to defibrillate hearts (Montgomery et al., 1954). However, it was not until 1955 that Lam and colleagues in the US administered an intraventricular injection of KCl (667 mEq/L) to induce cardiac arrest in hypothermic dogs (without bypass) (Lam et al., 1955). Lam's group subsequently abandoned the KCl method because of refractory ventricular fibrillation (VF) and myocardial damage during reperfusion and reanimation (Effler et al., 1956; Lam et al., 1957; Schaff et al., 1978; Shiroishi, 1999) (Figure 2). Lam also tried acetylcholine (10 mg/kg body wt) to induce arrest and while the heart would beat more forcefully during reanimation than when KCl was used, the incidence of VF was higher (Weirich, 1962). Lam also noted that O_2_ consumption of the potassium-arrested heart was lower than acetylcholine, implying it provided a more complete arrest (Lam et al., 1955).

In 1955 Melrose and colleagues, after corresponding with Lam's group, conjectured that the likely source of arrhythmias in Lam's KCl model was from the chloride. The London team performed the first cardioplegic arrest using a citrated form of potassium in a canine model of cardiopulmonary bypass. The “Melrose technic,” as it became known, used 2.0 ml of 25% solution of tri-potassium citrate added to 18 ml warm oxygenated whole blood in a syringe (9:1 blood: potassium ratio), and injecting directly into the aortic root of hypothermic dogs (25°C) (Melrose et al., 1955; Gerbode and Melrose, 1958). The heart arrested in seconds and remained electrically quiescent for 30 min and returned good function in the healthy dog. Melrose's group were the first to propose that high potassium-citrate may afford “elective reversible cardiac arrest” in human open-heart surgery (Melrose et al., 1955). Three years later, Gerbode and Melrose increasingly used potassium citrate to induce cardiac arrest in humans (Gerbode and Melrose, 1958).

Despite the early clinical excitement (Effler et al., 1956), the “Melrose technic” was abandoned in the late 1950s because it predisposed the heart to what Lam and colleagues had always suspected; refractory ventricular fibrillation, contractile dysfunction and cell death (Allen and Lillehei, 1957; Helmsworth et al., 1959; Nunn et al., 1959; Urschel et al., 1959; Wasserman et al., 1959; Willman et al., 1959; Bjork and Fors, 1961; Tyers et al., 1975). In 1962, Weirich wrote: “The use of the Melrose method of elective cardiac arrest induced by the single injection of a 2.5 percent potassium citrate-oxygenated blood solution has been abandoned by virtually all physicians who perform intracardiac operations.” (Weirich, 1962). It is curious why little or no mention was made by Melrose, Lam or early adopters about the possible mechanism of potassium citrate or potassium chloride arrest, given that K^+^-induced membrane depolarization was demonstrated by Woodbury in 1950 using microelectrodes in single cardiac fibers (Woodbury et al., 1950; Draper and Weidmann, 1951), by Burgen in isolated atria (Burgen and Terroux, 1953) and by Weidmann and colleagues in whole hearts (Niedergerke, 1956; Weidmann, 1956; Hoffman, 2002) (Figure 2). It appears that there were few collaborations between those studying surgical cardioplegia and researchers in the basic electrophysiological sciences. Importantly, while the “Melrose Technique” failed to reach efficacy, Melrose, Lam and their colleagues contributed enormously to the early history of cardiac surgery because they showed that surgical cardioplegia, as a strategy of buying biological time, was feasible.

Over the next 15 years, high potassium cardioplegia was placed on “clinical hold” by cardiac surgeons themselves as a result of poor clinical outcomes (Nunn et al., 1959) (Figure 1). Potassium-based solutions were eventually replaced by either: (1) hypothermic or normothermic ischemic arrest induced by aortic occlusion, (2) by intermittent aortic occlusion, or (3) by direct coronary artery perfusion (Kirklin et al., 1979; Mentzer et al., 2003). Shumway and associates proposed another modification using topical hypothermia as a “safeguard” to offset ischemic injury (Shumway et al., 1959). Unfortunately, these alternatives were no better than the “Melrose Technique,” which was highlighted by Cooley's irreversibly damaged “stone heart” (Cooley et al., 1975).

**Figure 3:**
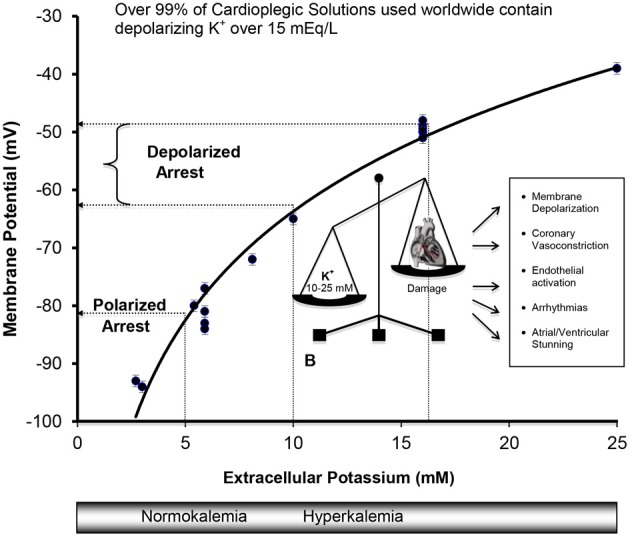
**Cell Membranes of the Atria, Purkinje, and Ventricular Myocytes *in vivo* behave as a K^+^ electrode over a range of extracellular K^+^ from 3 to 25 mM.** Data were obtained from isolated rat, rabbit, and guinea-pig hearts (Kleber, 1983; Masuda et al., 1990; Sloots and Dobson, 2010; Dobson and Jones, 2004; Dobson, 2010) and from isolated cells (Wan et al., 2000). The membrane potential φ (mV) = 27 ln [K^+^] − 126, *R*^2^ = 0.97, where [K^+^] is extracellular potassium concentration in mM. Membrane potentials (φ) were measured directly using potassium microelectrodes (Kleber, 1983; Masuda et al., 1990; Snabaitis et al., 1997; Wan et al., 2000) or calculated from Nerstian distributions of potassium (Dobson and Jones, 2004; Sloots and Dobson, 2010). The relation between potassium and membrane potential for ventricular muscle also agree well with microelectrode measurements on isolated human atrial muscle (Gelband et al., 1972), and for isolated purkinje fibers above 5.4 mM K^+^ bathed in Tyrode's solution (Sheu et al., 1980).

The field of cardioprotection in the 1960s was in desperate need of fresh ideas, innovation and improved outcomes. As a result, scientists and surgeons began to collaborate and, in the late 1960s and early 1970s, a second wave of clinical interest in potassium cardioplegia began to surface in Germany (Hoelscher, 1967; Kirsch et al., 1972; Bretschneider et al., 1975), the UK (Hearse and Stewart, 1974; Hearse et al., 1981) and the USA (Gay and Ebert, 1973; Kirklin et al., 1979; Buckberg, 1993) (see Figure 2). The collective body of experimental work was impressive and formed the basis of today's cardioplegia and organ preservation solutions (Cordell, 1995; Vinten-Johansen et al., 2000; Chambers and Hearse, 2001). Currently over 99% of cardioplegic and most preservation solutions contain K^+^ concentrations over 15 mEq/L (or mM), which depolarizes the myocardial cell membrane from a resting voltage of about −85 mV (~5 mM K^+^) to −50 mV (Dobson, 2010). The relationship between increasing extracellular potassium concentrations and membrane potential in the ventricle during cardioplegic diastolic arrest from isolated rat, rabbit, and guinea-pig hearts is shown in Figure 3. As we shall discuss below, for over four decades depolarizing potassium cardioplegia has been a double-edged sword, particularly after Tyers and colleagues demonstrated unequivocally that potassium, not citrate, was responsible for the clinical failure of “Melrose's method” (Tyers et al., 1975). Depolarizing potassium solutions remain the standard-of-care for cardioplegic arrest in cardiac surgery today.

## Five areas of concern with hyperkalemic cardioplegia

“Thus, although the use of elevated concentrations of potassium to induce the rapid depolarized arrest of the heart in diastole is by far the most widely used technique … it cannot be used haphazardly, has a number of disadvantages, and is not necessarily the best and most optimally protective.”

Chambers and Hearse (2001) p. 894

From a scientific standpoint, there are at least five (5) areas of concern with potassium concentrations of 10 mM and above (Dobson, 2010): (1) unnatural membrane voltages and cellular ionic imbalances during *globa*l ischemic arrest and *regional* ischemia during reperfusion; (2) coronary vasoconstriction of varying degrees leading to maldistribution of cardioplegia, loss of myocardial protection and possible vascular spasm; (3) activation the coronary vascular endothelium to become leaky, pro-inflammatory and promotes platelet aggregation; (4) post-operative arrhythmias and conduction disturbances; and (5) a higher incidence of low cardiac output from ventricular stunning.

### Adverse effects of hyperkalemia on membrane potential and Ca^2+^ loading

“In (heart) fibers which do not have pacemaker characteristics, the diastolic potential behaves as a K^+^ electrode and varies as expected by the Nernst relationship when the extracellular K^÷^ concentration is above 3 mM.”

Leonard S. Gettes (Gettes, 1976) p. 792

Hyperkalemia arrests the heart in diastole due to potassium's ability to reduce the availability of “open” Na^+^ fast channels, which are responsible for the rapid Phase O upstroke (depolarization) of the cardiac action potential (Kleber, 1983; Chambers and Hearse, 2001; Dobson and Jones, 2004). Cell membranes of the atria, Purkinje fibers, and ventricles behave like what Leonard S. Gettes termed “a potassium electrode,” meaning that the membrane potential during diastole is close to a Nernstian potassium potential (Gettes, 1976) where the sum of the net electrical and chemical concentration forces on the potassium ion is zero [ΔG (Al-Mehdi et al.)_out/in_ = 0] (Masuda et al., 1990; Veech et al., 1995). The Nernstian potential for the isolated rat heart over a range of 3–25 mM extracellular K^+^ can be calculated from the following relation where (Figure 3).

Membrane potential (φ mV) = 26 ln [K^+^, mmol/L] + 123 (Sloots and Dobson, 2010).

The close agreement between these Nernstian estimates calculated from intra- and extracellular K^+^ distribution (Sloots and Dobson, 2010), and direct K^+^ electrode measurements implies that the resting membrane potential in heart muscle is predominately a potassium equilibrium potential within the errors of measurement (i.e., behaves as a K^+^ electrode) (Gettes, 1976).

**Figure 4:**
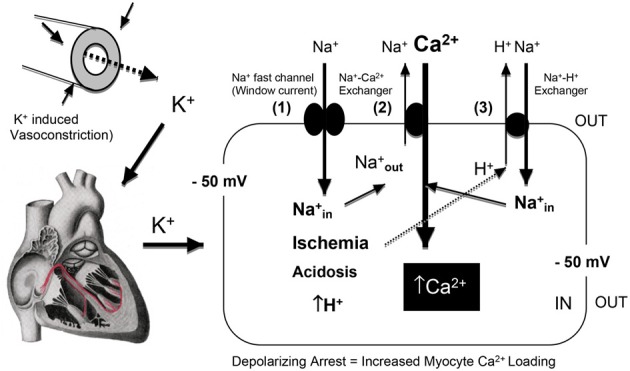
**A schematic of the effect of hyperkalemia and prolonged myocardial membrane depolarization on Na^+^ entry through the “window current” and the net influx of Ca^2+^ into the myocardial cell via the reversal of the Na^+^/Ca^2+^ exchanger [3Na^+^ ions are extruded in exchange for 1 Ca^2+^ entry (Bers and Despa, 2009) at a membrane potential of −50 mV (Baczko et al., 2003)].** Global or regional ischemia and metabolic acidosis impact further on Ca^2+^ loading, with increases in intracellular H^+^ further activating the Na^+^/H^+^ exchanger (Avkiran, 2001) resulting in 1Na^+^ ion being exchange for 1 H^+^ ion. The diagram was adapted from Bers and colleagues (Bers et al., 2003; Bers and Despa, 2006).

The clinical problems associated with prolonged membrane depolarization include Ca^2+^ loading of the myocyte and IR injury (arrhythmias, stunning, inflammation, necrosis, and apoptosis) (Suleiman et al., 2001). Depolarization-induced Ca^2+^ loading occurs first from Na^+^ entry through the voltage-dependent Na^+^ fast channels. At −50 mV (16 mM K^+^), despite ~3.5% availability of Na^+^ channels and only 0.1% of maximal Na^+^ conductance compared to −80 mV, the high sodium driving force (ΔG_Na+out/in_ = −15 KJmol^−1^) leads to Na^+^ entry via the small Na^+^ “window” current that remains open at these depolarized states (Bers et al., 2003). A rise in cell Na^+^ leads to a reversal of the voltage-dependent Na^+^/Ca^2+^ exchanger (3 Na^+^out: 1 Ca^2+^ in), with a resulting rise in intracellular Ca^2+^ (see Figure 4).

**Figure 5:**
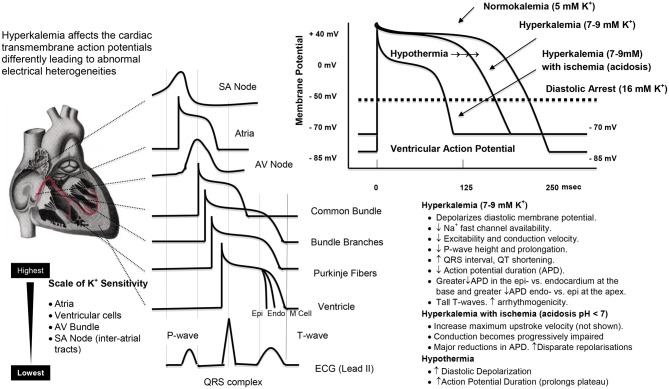
**A representation of the regional sensitivities in the heart to high potassium, and the effects of hyperkalemia, ischemia (acidosis), and hypothermia on the ventricular action potential.** The effects were obtained from the empirical data in rabbit ventricle and the ventricular modeling studies of Shaw and Rudy (1997) and Cimponeriu et al. (2001). The effects of hypothermia to prolong the action potential are from West et al. (1959), Lathrop et al. (1998) and Coraboeuf et al. (1998; Aoki et al., 1994).

Further Ca^2+^ entry can occur during Ischemia, acidosis and hypothermia. Global ischemia may lead to further membrane depolarization from localized increases in extracellular K^+^ (11 mM or higher) (Harris et al., 1954; Gettes et al., 1982; Kleber et al., 1987) and intracellular acidosis can activate the Na^+^/H^+^ exchanger (H^+^_out_ Na^+^_in_) and Ca^2+^ enters via the Na^+^/Ca^2+^ exchanger (Na^+^_out_ and Ca^2+^_in_) (Figure 4). These processes may be of reduced importance during surgical cardioplegia because metabolism (aerobic and anaerobic) has already been slowed by: (1) electrochemical arrest, and (2) hypothermia. However, if subendocardial regions of the left ventricular wall are not adequately perfused and protected during cardioplegic arrest (Brazier et al., 1977), regional differences in Ca^2+^ loading and ischemia-reperfusion damage may occur. Hypothermia is also known to exacerbate the effect of ischemia and Ca^2+^ loading (Sprung et al., 1995; Lathrop et al., 1998; Gaillard et al., 2000) and alter the properties of the ventricular action potential (Figure 5). The vasoconstrictive effects of hyperkalemia may further contribute to sub-optimal subendocardial protection (see next Section). In contrast, in pediatric cardiac surgery a very different “ischemic” scenario may exist where the baby heart is normally arrested with a “one shot” delivery (Allen, 2004). In this case, the heart cells are bathed in a cold, static ischemic and depolarized state, with myocardial protection becoming more compromised with time, particularly during complex corrective operations requiring longer cross-clamp times.

Following cold ischemic arrest, as mentioned earlier, Ca^2+^ loading also occurs during reperfusion. There are four time-sensitive periods of reperfusion; rewarming, reperfusing, reanimating and returning the heart to the general circulation. *Protecting the heart during the “4 R” periods is paramount to satisfactory clinical outcome* (Vinten-Johansen and Nakanishi, 1993; Aoki et al., 1994; Liu et al., 1994; Cascio et al., 1995; Wilde and Aksnes, 1995; Stowe et al., 2000). As the heart moves from a cold depolarized state to a warm polarized state, it is electrically and metabolically challenged because of transmural and regional voltage heterogeneities which have to rapidly return to their “normal” operating limits to ensure a coordinated wave of depolarization from the SA node, atria, AV node to the ventricles (see Figure 5). The effects of hyperkalemia cardioplegia, combined with the effects of ischemia, acidosis and hypothermia, predisposes the heart to electrical instability during reperfusion which can lead to arrhythmias (Section Adverse Effects of High K^+^ on Post-operative Arrhythmias and Conduction Disturbances) and post-operative left ventricular pump dysfunction (Section Adverse Effects of High K^+^ on Cardiac Output and Stunning) (Tsutsumi et al., 1983; Flack et al., 1992). These effects of IR injury will be further exacerbated in older, sicker patients undergoing longer cardiopulmonary bypass times, or in complex pediatric congenital corrective operations, which pose a serious challenge to current hyperkalemia myocardial protection strategies.

### Adverse effects of hyperkalemia on coronary vasoconstriction

“Cardioplegia, whether blood or crystalloid, is also intrinsically associated with functional changes in the coronary vasculature that add to the effects of cardiopulmonary bypass. This may be observed after even routine cardiac cases, with up to 8% of patients having coronary artery spasm manifested by temporary ST segment elevations on ECG after surgery, and an even greater proportion exhibiting myocardial contractile dysfunction that usually peaks 4–6 h postoperatively. These phenomena may be seen in virtually any patient regardless of age, and irrespective of the presence of atherosclerotic coronary artery disease or other risk factors for endothelial dysfunction.”

(Ruel et al., 2004) p. 1002

Potassium-induced coronary vasoconstriction may result in loss of cardioprotection by limiting cardioplegia flow and distribution to the diseased myocardium (Leicher et al., 1983; Sellke et al., 1996; Aronson, 1999). High potassium is also linked to coronary spasm in adults, with or without hypertension (Ruel et al., 2004) and in pediatric patients (Nomura et al., 1997). Experimentally, high potassium solutions have been used for over 40 years as a tool to induce *maximal* vasoconstriction in aortic ring or coronary artery conduit preparations (Norton and Detar, 1972; Kuo and Chancellor, 1995; Sellke et al., 1996; Chong et al., 2001; Kerendi et al., 2004).

**Figure 6:**
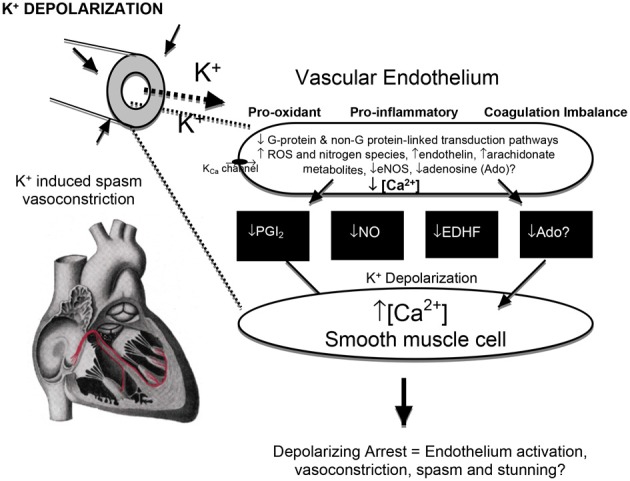
**The effect of high potassium on the endothelium and smooth muscle interactions, vasoconstriction, and possible injury.** Membrane K^+^ depolarization decreases the driving force for Ca^2+^ entry into endothelial cells through Ca^2+^-activated K^+^ channels (Coleman et al., 2004), increases voltage sensitive endothelial NADPH oxidases (and other oxidants) (Sellke et al., 1993; Li and Shah, 2004) which can lead to vascular smooth muscle contraction and vasoconstriction (van Breemen et al., 1997) from a reduced availability of endothelial-derived vasodilators [prostacyclin (PGI_2_), nitric oxide (NO), non-NO/PGI_2_ relaxation factors (EDHF's) and adenosine] (He, 2005; Wu et al., 2005; Yang and He, 2005). Hyperkalemia in cardioplegia has been linked to loss of ACh-dependent relaxation, which may be exacerbated by ischemia and hypothermia (Tyers, 1997; Parolari et al., 2002; Yang and He, 2005).

Vasoconstriction can result from direct depolarization of vascular smooth muscle or the underlying endothelium (Figure 6). Quignard and coworkers showed that cardioplegic potassium concentrations (15 mM) depolarized vascular smooth muscle membranes from −53 to −45 mV in guinea-pig carotid and porcine coronary arteries, which led to contraction (Quignard et al., 1999). Casteels and colleagues further showed that the minimal depolarization to evoke contraction of smooth muscle cells isolated from the pulmonary artery was at 9.0 mM KCl (Casteels et al., 1977). Vasoconstriction can also occur from the underlying vascular endothelium, which is electrically connected to smooth muscle (Seiden et al., 2000; Han et al., 2009) (Figure 5). Coronary vascular tone (relaxation/constriction) is regulated by a delicate balance between endothelial vasconstrictive and vasorelaxive factors, which in turn modulate “end-effector” potassium channels (K_Ca_, Kv, and K_ATP_ channels) and other channels located in underlying vascular smooth muscle of the vessel wall (Figure 6) (Brayden, 1996; Han et al., 2009; Dick and Tune, 2010). Furchgott and Zawadzki were the first to show a link between hyperkalemia-induced vasoconstriction and impairment of endothelium-derived nitric oxide (NO) release, and other relaxation factors (Furchgott and Zawadski, 1980). Other relaxation factors include prostacyclin (PGI_2_), endothelium-derived hyperpolarization factor (EDHF) and adenosine and/or conducted hyperpolarization processes which link the endothelium to vascular smooth muscle function (He, 2005; Wu et al., 2005; Yang and He, 2005). He and colleagues in 2005 showed, for example, that incubating porcine small resistance coronary arteries in either St. Thomas's solution or Celsior preservation solutions resulted in a significant potassium-induced depolarization and loss of endothelial-derived hyperpolarizing factor (EDHF)-mediated function (Wu et al., 2005; Yang and He, 2005). The endothelium-derived vasoconstrictor *Endothelin*-1 may contribute to vasoconstriction (Feng et al., 2010), as it is involved in ischemia-reperfusion injury, spasm and transient left ventricular dysfunction following hyperkalemic arrest (Knothe et al., 1992; Dorman et al., 2000).

Magnesium is often added to cardioplegia to reduce intracellular Ca^2+^ loading during ischemia (Nakaigawa et al., 1997; Ichiba et al., 1998; Murphy, 2000; Mubagwa et al., 2007) and to counter K^+^-induced vasoconstriction (Matsuda et al., 1999; Yang and He, 2005). Colder temperatures also offset the vasoconstriction effects of high potassium (Chiavarelli et al., 1982), however, it can lead to local and systemic inflammation, coagulation disturbances and tissue edema (Mezzetti et al., 1995; Torracca et al., 1996; Gaillard et al., 2000; Torchiana et al., 2000; Ravishankar et al., 2003). Thus, while hypothermia partially mitigates the potassium constrictor property, it may not provide optimal myocardial protection in cardiac surgery.

### Adverse effects hyperkalemia on myocardial endothelial function

“Modern cardiopulmonary bypass and cardioplegic arrest techniques remain associated with an acute transcriptional response of hundreds of genes that primarily effect the largest organ of the body, the endothelium, as well as other constituents of blood vessels.”

(Ruel et al., 2004) p. 1002

The endothelium sits at the geographical nexus between the blood and myocardium and has an exceptionally high surface area of ~35 m^2^ in the adult heart for only ~3% of the total myocardial volume (Hearse and Stewart, 1974; Verma and Anderson, 2002). Along with high potassium's vasoconstrictive and spasmodic effects (discussed above), K^+^ levels as low as 10 mM K^+^ have been linked to endothelial injury including vascular depolarization, inflammation, neutrophil adherence, leaky junctions, platelet activation, pro-oxidant production, and impaired coagulation status (Jellinek et al., 1981; Mankad et al., 1991, 1997; Nilsson et al., 1993; Sellke et al., 1993; Parolari et al., 2002; Verma and Anderson, 2002; Perrault and Carrier, 2005; Higashi et al., 2009) (Figure 6).

As early as 1981 Jellinek and colleagues showed that high potassium (>30 mM) led to structural changes in endothelial cells such as intracellular vacuolization and blebbing with vacuole formation, and that these deleterious effects were reduced with hypothermia (Jellinek et al., 1981). In the same year, Carpentier et al. (1981) reported that depolarizing potassium cardioplegia markedly reduced the viability of human endothelial cells and fibroblasts after several hours of incubation. Hoover and colleagues further demonstrated in vein grafts exposed to potassium solution that there was an aggressive adventitial fibrotic reaction compared to a control normokalemic Ringer's solution (Hoover et al., 1981). In 1983, Olinger and others showed in their primate model of graft atherogenesis that prolonged exposure of the cephalic vein grafts to hyperkalemic crystalloid cardioplegic solutions (27 mM KCl) resulted in increased lipid uptake and cholesterol deposition in the subintimal space 12 weeks after surgery (Olinger et al., 1983). It is possible that high potassium lead to intimal damage and loss of patency in the 16–25% of saphenous vein conduits that occur in the first post-surgical year, and 10% of occlusions in radial artery grafts (Goldman et al., 2004; Magee et al., 2008; Buxton et al., 2009). This association between depolarizing hyperkalemia and graft failure requires further investigation.

While the mechanisms of high K^+^-induced endothelial damage are controversial, it appears that membrane depolarization is a first step of injury and activation *and oxidant production* (Figure 6). Membrane *depolarization* increases voltage sensitive endothelial NADPH oxidases (and other oxidants) and contributes to oxidative injury and endothelial activation (Sellke et al., 1993; van Breemen et al., 1997; Li and Shah, 2004). Many years ago, Yacoub and colleagues reported that increasing the concentration in St. Thomas's solution (Plegisol®) or Bretschneider's (HTK, Custodiol®) solution from 20 to 30 mM played a critical role in causing endothelial damage, which was assessed as a loss of endothelium-dependent vasodilation (Mankad et al., 1991). In 2008, Radovits and colleagues stored isolated rat aortic rings for 24-hour in cold traditional Bretschneider's solution [HTK, Custodiol®], and reported endothelial dysfunction (Radovits et al., 2008). In 1993, Sellke and others showed that coronary endothelial dysfunction after ischemic cardioplegic arrest and cardiopulmonary bypass, and it was also involved with the production of oxygen-derived free radicals (Sellke et al., 1993).

Endothelial membrane depolarization is known to promote platelet adhesion and neutrophil activation and inflammation (Niu et al., 1996; Halkos et al., 2004; Li and Shah, 2004). Interestingly, as early as 1987 Ishikawa and Sasakawa showed that increasing K^+^ led to increasing depolarization of platelets, and that aggregation induced by ADP or collagen was enhanced by depolarization with increasing extracellular [K^+^] (Ishikawa and Sasakawa, 1987). In 2003, Krotz and colleagues similarly showed that endothelial membrane depolarization promoted platelet aggregation through increased superoxide production (O^−^_2_) and inactivation of endothelial ectonucleotidases, and this effect was abolished by membrane hyperpolarization (Krötz et al., 2003). In 2005, Matsuzaki et al., further reported membrane depolarization-induced ROS generation when rat and mouse endothelial cells were switched from a normokalemic (5.9 mEq K^+^) to a hyperkalemic bathing solution (24 mEq K^+^) (Matsuzaki et al., 2005). Platelet aggregation and neutrophil adhesion are thought to be key events in inflammation and acute vascular thrombosis, particularly when activated platelets come into contact with the subendothelial matrix, like collagen (Siess, 1989).

High K^+^ also promotes *tissue oxidative stress*, which may lead to myocardial ischemia-reperfusion injury and exacerbate inflammation (Figure 6). Al-Mehdi and colleagues have employed high potassium to convert lung cells from a normal to pro-oxidant state (Al-Mehdi et al., 1997, 1998). These workers showed in the intact lung or reconstituted cell systems that high potassium triggered a depolarization-linked activation of endothelial NADPH oxidase and extracellular generation of O^−^_2_ (Al-Mehdi et al., 1997, 1998). In 2000, Sohn and others also used high potassium (90 mM) to depolarize human endothelial cells, and found that the extent of depolarization of the membrane was directly responsible for the production of superoxide (O^−^_2_), and that the effect was inhibited in the presence of a hyperpolarizing K_ATP_ channel opener (HOE 234) (Sohn et al., 2000). Furthermore, pretreatment with the tyrosine kinase inhibitor genestein completely abolished the K^+^ depolarization-induced production of O^−^_2_, indicating an involvement of tyrosine phosphorylation (Sohn et al., 2000) (Figure 5). Thus, there is a strong association between high potassium cardioplegia, endothelial activation, and oxidant stress which may lead to post-operative cardiac and vascular dysfunction.

### Adverse effects of hyperkalemia on post-operative arrhythmias and conduction disturbances

“Potassium, calcium, sodium, and magnesium play a role in the genesis of experimental arrhythmias. In the clinical setting, however, altered K^+^ concentration is responsible for the vast majority of such arrhythmias. This is true because within the range of levels of various electrolytes encountered in clinical disorders, K^+^ is the electrolyte most likely to alter the electrophysiologic properties of the heart.”

Fisch C (1973) (Fisch, 1973)

Early postoperative arrhythmias are a frequent complication of adult and pediatric cardiac surgery (Harris et al., 1954; Ebert et al., 1962; Flack et al., 1992; Cohen et al., 1993; Yau et al., 1993; Gaillard et al., 2000; Valsangiacomo et al., 2002). In 1980, Ellis showed that higher potassium concentrations in cardioplegia in humans resulted in a higher number of ventricular arrhythmias following cross-clamp removal, and this was worse at lower temperatures (Ellis et al., 1980). Post-operative arrhythmias can lead to left ventricular dysfunction, hemodynamic deterioration and costly treatment regimens amounting to billions of dollars per annum in the USA alone (Aranki et al., 1996; Mathew et al., 2004; Steinberg, 2004; Echahidi et al., 2008; Anselmi et al., 2009). Arrhythmias are also a major cause of morbidity and mortality in pediatric patients (Lan et al., 2003), where the overall incidence of arrhythmias can be as high as 48% (Pfammatter et al., 2002; Valsangiacomo et al., 2002). We are unaware of any studies having investigated the role of hyperkalemic cardioplegia on the incidence of early post-operative arrhythmias in the pediatric patient.

High potassium-linked arrythmogenicity is related to the creation of abnormal regional and transmural electrical heterogeneities required for the normal conduction of the heart (Figure 5). It has been known for over 40 years that the atria are the most sensitive to high K^+^ (de Mello and Hoffman, 1960), followed by ventricular cells, His cells, SA nodal cells, and inter-atrial tracts (Ettinger et al., 1974) see (Figure 5). That the SA node is more resistant to increasing K^+^ explains why nodal activity persists after early cardioplegic arrest of the atria and ventricles. Thus, differences in regional and wall potassium sensitivities, combined with localized K^+^ changes from cold ischemia and diastolic membrane voltage changes, may give rise to new onset arrhythmias following cardiac surgery. Tsutsumi and colleagues further showed differences in K^+^ sensitivity across the wall of the ventricle at the base and near the apex of the heart (Tsutsumi et al., 1983). Higher K^+^ resulted in greater decreases in repolarization times in the basal epicardium compared with the endocardium, and a greater decrease at the endo vs. epicardium near the apex (Tsutsumi et al., 1983). Abnormal times for ventricular repolarization (Figure 5), along with local conduction slowing, may be key factors in *predisposing* the heart to arrhythmias during reanimation (Ettinger et al., 1974; Tsutsumi et al., 1983).

Depolarizing potassium can also lead to conduction (SA, AV, and Bundle of His) disturbances (Taggart et al., 2000). From the early 1980s, there were many groups investigating the failure of standard high potassium cardioplegia and hypothermic techniques to protect the heart's conduction system during cardiac surgery (Ellis et al., 1980). Ferguson and colleagues using multiple bipolar intracardiac and unipolar intramural electrodes reported the presence of electrical activity in the lower atrial septum, the atrioventricular node-His bundle complex, and in ventricular myocardium during hyperkalemic cardioplegic arrest that interestingly could not be detected visually or on the limb-lead electrocardiogram (Ferguson et al., 1986). Magilligan and colleagues suggested additional local cooling was needed at specific sites to reduce the incidence of conduction disturbances, block and junctional rhythm and low ventricular output following reperfusion (Magilligan et al., 1985; Ferguson et al., 1986). In summary, regional hyperkalemia and sensitivities (Figure 5), may induce regional refractroriness and local conduction slowing, which predispose the heart to post-operative arrhythmias.

Could the effect of hyperkalemia, hypothermia, and electrical disturbances contribute to post-operative atrial fibrillation (AF)? As mentioned in the introduction, AF is an early comorbid event with an incidence of around 30% after CABG and as high as 69% after more complex procedures (Almassi et al., 1997; Steinberg, 2004; Scherr et al., 2006). At present it is unclear whether high K^+^ in cardioplegia is directly linked to post-operative AF (Mathew et al., 2004; Steinberg, 2004; Echahidi et al., 2008; Anselmi et al., 2009), as similar incidences occur following “on-pump” and “off-pump” where no hyperkalemic cardiopelgia is used (Hosokawa et al., 2007). However, it is possible that the underlying causes of AF in both types of surgery have direct and indirect roles for depolarizing K^+^ in a setting of ischemia-reperfusion, endothelial dysfunction and inflammation (Vinten-Johansen et al., 2007a). In “on-pump surgery,” AF could be exacerbated from high K^+^ in the surgical cardioplegia, and in “off-pump” surgery from rises in local extracellular K^+^ from multiple bouts of regional ischemia during occlusion of the target vessel, and from the compounding effect of acidosis which can activate endothelial cells to produce damaging oxidants and amplify the inflammatory process (Kitagawa and Johnston, 1985; Al-Mehdi et al., 1997). Regional ischemia can lead to increases in extracellular K^+^ of over 20 mM, and Miura's group showed that these changes are linked to non-uniform rises in intracellular Ca^2+^ and sustained arrhythmias (Miura et al., 2012). In 2008, Kon and colleagues also reported a strong correlation during “off-pump” cardiac between myocardial ischemia and gradients of transcardiac inflammation markers (TNF-alpha, IL-8) and thrombosis (thrombin generation-F1.2, contact activation pathway-FXII-a, platelet derived microparticles) (Kon et al., 2008). This data suggests that even brief episodes of ischemia-reperfusion during off-pump surgery may cause local injury currents in the atria, which may be significant because the atria are the most sensitive to high K^+^ (Figure 5). In 2009, Tran and colleagues also reported a link between plasma K^+^ during cardiac surgery and post-operative atrial fibrillation (Tran et al., 2009). Thus, hyperkalemia, whether imposed on the heart from depolarizing K^+^ in surgical cardioplegia or from bouts of regional ischemia during beating heart surgery, may be linked functionally to membrane depolarization and an activated endothelium and inflammatory response, which collectively promote the generation of atrial (and ventricular) arrhythmias. Further studies are required to test this hypothesis.

### Adverse effects of hyperkalemia on cardiac output and stunning

“There is clinical evidence that myocardial stunning is a frequent sequela of surgical global ischemia, despite our modern techniques of myocardial protection. The ubiquitous usage of hyperkalemic depolarizing solutions in all forms of cardioplegia may be partly responsible for this phenomenon because of the known ongoing metabolic requirements and damaging transmembrane ionic fluxes that occur at depolarized membrane potentials.”

Damiano RJ Jr, Cohen NM. (Damiano and Cohen, 1994)

Left Ventricular dysfunction is a key determinant of patient outcome following cardiac surgery (Algarni et al., 2011). One form of post-operative LV dysfunction is myocardial stunning or the loss of post-operative contractility from IR injury *without cell death* (Braunwald and Kloner, 1982; Kloner et al., 1994). The phenomenon was first described experimentally in 1975 by Heyndrickx and colleagues, who reported an abnormal, depressed ventricular function after regional ischemia in dogs despite normal return of coronary blood flow and ECG waveform (Heyndrickx et al., 1975). It is manifest as a decrease in cardiac output typically lasting 2–4 h post-operatively (Gillies et al., 2005), and can persist for longer periods without apparent signs of infarction or other serum injury markers (Troponins or CKMB) (Kloner and Jennings, 2001). Stunning occurs in about 10% of adults following cardiac surgery (Weisel, 1993; Vaage and Valen, 1993; Mangano, 1995; Spinale, 1999; Flack et al., 2000; Chang et al., 2002; Anselmi et al., 2004) and in 10–15% of pediatric patients (Parr et al., 1975; Booker, 1998; Ravishankar et al., 2003). Stunning may contribute to low cardiac output syndrome (LCOS) in adults (Algarni et al., 2011) and pediatric patients (Weisel, 1993; Booker, 1998; Spinale, 1999; Wessel, 2001; Allen, 2004; Jones et al., 2005). The incidence of LCOS occurs int 3–14% of adult CABG patients and is associated with a 10-fold to 17-fold increase in mortality (Algarni et al., 2011), and in around 25% of pediatric cardiac surgery patients (Jones et al., 2005). The classic features of LCOS include tachycardia, oliguria, poor peripheral perfusion, and low blood pressures (Jones et al., 2005). Importantly, a low ejection fraction (EF) is a poor predicator of overall cardiac reserves and does not predict LCOS (Zaroff et al., 1994), however, a low EF plus inadequate cardioprotection is associated with LCOS and possible stunning (Zaroff et al., 1994; Aronson, 1999).

Hyperkalemic cardioplegia may contribute to myocardial stunning and LCOS from the effect of high potassium and IR injury on Ca^2+^ loading, vasoconstriction, reanimation arrhythmias, and endothelial dysfunction (Figures 3–6) (Booker, 1998; Allen, 2004; Amark et al., 2005). In animal models cold hyperkalemic cardioplegia, and the transition from hypothermia to normothermia, before cross-clamp removal is also a factor linked to stunning (Przyklenk et al., 1994; Spinale, 1999; Eberhardt et al., 2000; Ambrosio and Tritto, 2001). An area of particular interest in stunning is the release of catecholamines from adrenergic nerve terminals leading to further transient Ca^2+^ loading (Lubbe et al., 1992; Paterson et al., 1993; Vatner and Vatner, 1998; Lameris et al., 2000; Vittone et al., 2006), and the production of oxidants and oxygen-derived free radicals from injured myocytes and activated endothelial cells exacerbating inflammation and coagulation imbalances (Bolli et al., 1988; Carden and Granger, 2000; Kloner and Jennings, 2001; Zweier and Talukder, 2006). The direct and indirect roles of high potassium as part of the underlying pathophysiology of myocardial stunning warrant further investigation (Algarni et al., 2011).

## Five decades of searching for alternatives to depolarizing potassium

“Nothing is new except arrangement.”

Will Durant (1885–1981).

For over five decades, surgeons and scientists have acknowledged the adverse effects of high potassium cardioplegia (Chambers and Fallouh, 2010) (Figure 2). After a 10 year “moratorium” beginning in the late 1950s, Bretschneider and colleagues began to test alternatives, and in 1967 they hypothesized that dramatically reducing sodium (from ~130 mM to 12 mM) and keeping Ca^2+^ low (near zero), and reducing potassium in the presence of the Class 1 antiarrhythmic agent procaine (0.2%) may offer a safer and effective way to inhibit the action potential and arresting the heart (Reidemeister et al., 1967). In the same year, Hoelscher tested procaine as a non-depolarizing arresting agent in rabbit hearts, and confirmed that the “Melrose technic” using potassium citrate was pro-arrhythmic and caused severe myocardial damage (Hoelscher, 1967). In 1972, Kirsch proposed another cardioplegia formulation containing procaine, magnesium aspartate, and sorbital with apparently good clinical results (Kirsch et al., 1972). However, in 1977 Jynge et al. evaluated the components of the Kirsch and Bretschneider solutions using dose response curves, and compared the efficacy with their own home-grown St. Thomas' Hospital solution with 15 mM KCl and 16 mM MgCl_2_ (Jynge et al., 1977). Using the isolated Langendorff rat heart model, they concluded that the St Thomas Hospital solution had superior in functional recovery (Jynge et al., 1977). Jynge and colleagues further cautioned the field that until the mechanisms underlying ischemic damage were better understood, it would be unwise to recommend “the use of solutions containing extremes of concentration or solutions devoid of ions normally found in the extracellular fluid” (Jynge et al., 1977). St Thomas' Solution was introduced clinically in 1975.

In the 1970s the science of cardioplegia began to expand and diversify. In 1975 Tyers and associates introduced tetrodotoxin as another cardioplegia agent in the isolated rat heart (Tyers et al., 1975). Tetrodotoxin was first shown in 1960 by Narahashi to polarize the cell membrane, presumed by blocking sodium channels (Narahashi et al., 1960; Narahashi, 2008), and in 1997 Chambers and colleagues extended Tyers studies (Snabaitis et al., 1997). In 1983, Leicher and colleagues also tested blood cardioplegia using (1) 1.5 mM lidocaine alone, (2) 1.5 mM lidocaine with 30 mM potassium, and (3) 30 mM potassium alone, and they concluded that high K^+^ cardioplegia causes marked vasoconstriction, and impairs cardioplegic delivery and distribution (Leicher et al., 1983) (Figure 2). As a primary cardioplegic agent, lidocaine alone has not translated into surgical practice (Yamaguchi et al., 2007), however, the drug is commonly used at lower concentrations with or without magnesium during reperfusion to reduce ventricular arrhythmias (Baraka et al., 1993; Kanchi et al., 2004; Yamaguchi et al., 2007).

Another non-depolarizing cardioplegic agent was adenosine. In 1989, Opie and colleagues tested 10 mM adenosine in the isolated rat heart (Schubert et al., 1989). Belardinelli had previously shown that 50 μM adenosine inhibited isolated rabbit SA node pacemaker cells by hyperpolarizing the membrane by ~12 mV (Belardinelli and Giles, 1988). Opie and colleagues reported that high doses of adenosine induced rapid arrest but delayed post-ischemic recovery, and after assessing all the data they advocated switching back to depolarizing potassium cardioplegia with adenosine as an adjunct (De Jong et al., 1990). This work formed the basis for Phase I and II clinical trials in the late 1990s (Mentzer et al., 1999). Unfortunately, the trials showed no significant cardioprotective advantage of having adenosine present in hyperkalemic blood cardioplegia (Mentzer et al., 1997, 1999; Cohen et al., 1998; Vinten-Johansen et al., 2003). In 2007, Jacobsen and colleagues have shown that 1.2 mM adenosine instead of supranormal potassium in cold-crystalloid cardioplegic solution improved cardioprotection in the porcine model of cardiopulmonary bypass (Jakobsen et al., 2007). More recently, the same group showed in humans that the adenosine cardioplegia resulted in shorter times to arrest (11 vs. 44 s, *P* < 0.001) and lower postoperative atrial fibrillation (19 vs. 54% *p* = 0.01) compared with high potassium cardioplegia (20 mM) (Jakobsen et al., 2013). The discrepancy between Jacobsen's and previous adenosine cardioplegia studies (Schubert et al., 1989; Mentzer et al., 1999; Vinten-Johansen et al., 2003; Ahlsson et al., 2012) may be explained by their addition of local anesthetic (procaine) (Vinten-Johansen and Dobson, 2013). Dobson and colleagues have shown that adenosine plus a local anesthetic confers superior protection than adenosine alone (Dobson and Jones, 2004) (see Section Adenosine and Lidocaine (AL): A More Natural Way to Arrest, Protect, and Preserve the Heart). Dobson chose lidocaine because as a Class 1B anti-arrhythmic it shortens repolarization time, whereas procaine (Class 1A) prolongs repolarization time (Opie and Gersh, 2008), which may cause post-operative reanimation irritability (Vinten-Johansen and Dobson, 2013).

In the 1990s, ATP-sensitive potassium (K_ATP_) channel openers were another class of drugs that raised the interest of surgeons as alternatives to hyperkalemia (Figure 1). Following Noma's discovery of the K_ATP_ channel in heart in 1983 (Noma, 1983), a number of K_ATP_ openers were subsequently developed (e.g., pinacidil, aprikalim, nicorandil, and cromakalim), and they shared the property of hyperpolarizing the cell membrane of a number of tissues, including myocardium (Nichols and Lederer, 1991). At high concentrations, K_ATP_ channel openers prevented the Na^+^ fast channels from reaching their threshold to open and thereby arrested the heart in asystole (Chambers and Hearse, 2001). Initially there was great excitement about the possibility of K_ATP_ openers as primary non-depolarizing cardioplegic agents (Cohen et al., 1993, 1995; Lopez et al., 1996; Jayawant et al., 1997; Jayawant and Damiano, 1998). Unfortunately, like many new drugs, their application did not translate to clinical safety and efficacy because they failed to protect the myocardium, and many were pro-arrhythmic during reperfusion (Chi et al., 1990; Chambers and Hearse, 1999). The field had come full circle; non-depolarizing agents such as procaine and lidocaine, hyperpolarizing agents such as adenosine and K_ATP_ openers, once again became servants to high potassium concentrations. They were principally experimentally and in some cases clinically used as adjuncts in hyperkalemic cardioplegic solutions and longer-term preservation solutions, rather than the primary arresting agents (McCully and Levitsky, 2003; Diodato et al., 2005; Mizutani et al., 2006).

Other arresting agents have been experimentally examined and reexamined over the past 50 years, including acetylcholine (Lam et al., 1958), calcium antagonists (verapamil, nifedipine, and diltiazem) (Robb-Nicholson et al., 1978), high concentrations of magnesium (Kirsch et al., 1972), other sodium channel blockers (Snabaitis et al., 1997), 2,3-Butanedione monoxime (Chambers, 2003) and opioids (Bolling et al., 1997). More recently, non-depolarizing esmolol-HCl cardioplegia has been suggested as a possible alternative to high potassium (Warters et al., 1998; Chambers, 2003). Esmolol-HCl is an ultra-short-acting beta-blocker with a half-life of about 10 min, and at high concentrations can both arrest the heart and attenuate myocardial ischemia-reperfusion injury (Geissler, 2002; Chambers, 2003). Although Chambers showed that multidose infusions (2 min given every 15 min) of a 1.0 mmol/L esmolol solution arrested and protected isolated crystalloid-perfused rat hearts for up to 90 min at normothermic temperatures (Chambers, 2003), earlier work by Deslauriers and colleagues (Ede et al., 1997) reported esmolol-related increases in left ventricular diastolic pressure and pulmonary arterial pressures during arrest, and a difficulty in weaning the pigs off bypass (Ede et al., 1997). Further work is required to test the surgical applications of esmolol in the clinical setting.

## Adenosine, lidocaine, and Mg^2+^ (ALM™): a more natural way to arrest, protect and preserve the heart

“For any question in science, there is one animal best suited to study it”

August Krogh [quoted from Krebs (1975)].

### Lessons from natural hibernators

In the late 1990s, one of us (GPD) asked the question: “Could the human heart be pharmacologically modified to operate more like the heart of a natural hibernator during cardiac surgery?” (Dobson, 2004). The question taps into hundreds of millions of years of animal adaptations from Nature's own laboratory with the goal of developing new strategies and therapeutics to treat human disease. Wilfred Bigelow used the “August Krogh” principle in 1950 when he first introduced therapeutic hypothermia into cardiac surgery by recalling his experience as a boy in Canada and natural hibernators dropping their core temperatures to a few degrees above ambient (Bigelow, 1984). Bigelow wrote: “I became aware that surgeons would never be able to correct or cure heart conditions unless they were able to stop the circulation of the blood through the heart, open it, and operate in a bloodless field under direct vision” … “One night I awoke with a simple solution … *cool the whole body, reduce oxygen requirements*, *and interrupt the circulation and open the heart”* (Bigelow, 1984; Sealy, 1989).

With respect to slowing the heart, natural hibernating animals (or summer estivators) do not flood their heart cells with high K^+^ and depolarize their cell membranes as they enter torpor, hibernation or estivation (Willis et al., 1971; Al-Badry and Taha, 1983; Wang et al., 2002; Dobson, 2004). As temperature drops from 35 to 10°C, myocytes from the rat depolarize from about −80 to −45 mV see (Wang et al., 2002), whereas myocytes from the hibernating ground squirrel can be supercooled to −5°C and defend their membrane potential at around −60 mV (Wang and Zhou, 1999). Furthermore, natural hibernators do not vasoconstrict their coronary arteries with depolarizing K^+^, and potentially compromise blood flow distribution and myocardical protection during hibernation (Sjöquist et al., 1986; Burlington et al., 1989; Kudej and Vatner, 2003; Dobson, 2004). Studies on woodchucks (*Marmota monax*) have shown that coronary blood flow was maintained despite an 84% fall in cardiac output during hibernation relative to the awake state (Kudej and Vatner, 2003). *Thus*, *from the lessons of natural hibernators, the way surgeons have imposed depolarized, low energy states on the human heart and coronary vasculature using high K^+^ is unphysiological on at least two accounts*: (1) hibernators reduce their metabolism without imposing a depolarizing insult on their cells, and (2) the coronary arteries of hibernators are left wide open despite a low-energy torpid state of the heart.

### The AL(M) polarizing concept

**Figure 7:**
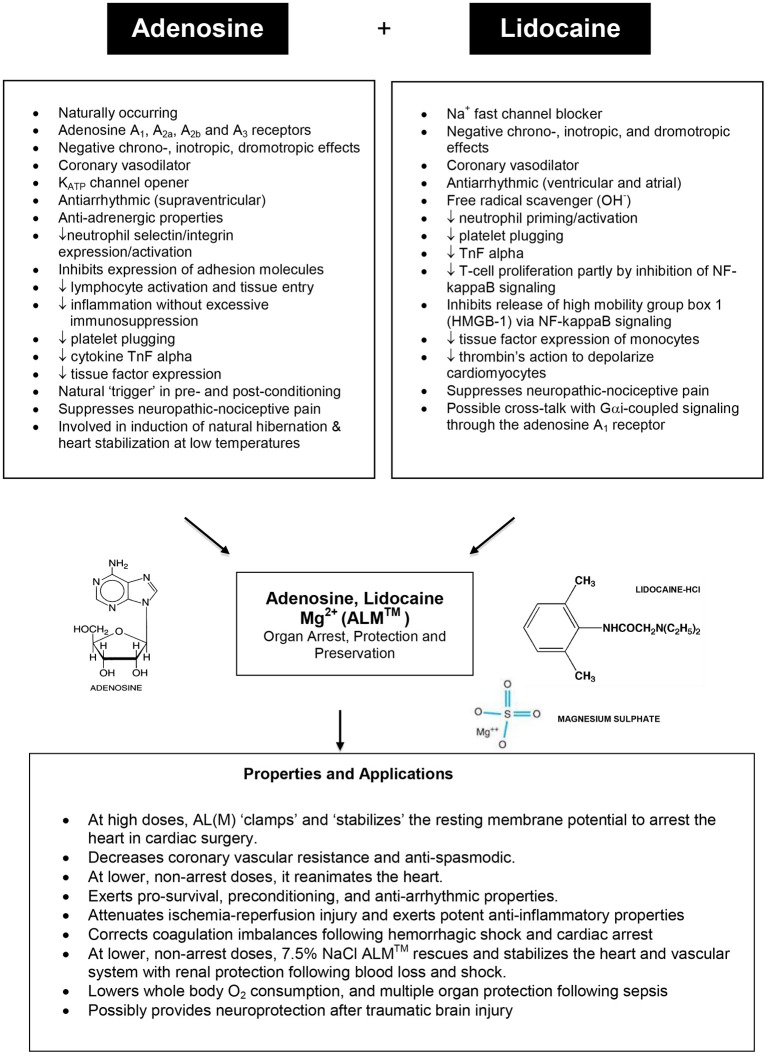
**Summary of the major properties and structures of adenosine and lidocaine (with Mg^2+^), and their potential mechanisms of action for heart arrest, protection and preservation (see text for details)**.

Down-regulating the heart during hibernation may involve multi-modulation of Na^+^ channel activity and associated innate central nervous system-linked-cell preconditioning mechanisms with the production of K_ATP_ channel openers or O_2_ demand-lowering agents by endogenous agents that are released during low oxygen transition states such as naturally occurring adenosine. Adenosine has been shown to be important in priming the brain for entrance into hibernation in the arctic ground squirrel (Jinka et al., 2011). The two drugs chosen to mimic the hibernating heart and examined 10 years ago from a long list of energy lowering compounds, K^+^_ATP_ openers, and Na^+^ blockers were adenosine and lidocaine (see Figure 7).

The AL polarizing hypothesis was formulated in 1998 and based on the following reasoning. If the *voltage*-*amplitude* (Phase O upstroke) of the cardiac action potential was reduced to its diastolic baseline by blocking the inward Na^+^ fast current (y-axis), and if the *action potential duration* of the atria, Purkinje fibers, and ventricles were reduced by opening K_ATP_ channels (x-axis) (see Figure 5), the heart should arrest at its “resting” diastolic membrane potential of −80 to −85 mV (Dobson, 2004). There would be no need for depolarizing potassium to induce electrochemical arrest. Along with adenosine opening K_ATP_ channels (Terzic et al., 1995) and lidocaine blocking Na^+^ fast channels (Josephson, 1988; Moller and Covino, 1988; Zamponi et al., 1993; Opie and Gersh, 2008), the additional benefit of arresting (and non-arresting) concentrations of A and L is that they both rapidly and reversibly slow heart rate (negative chronotropism) (Drury and Szent-Gyorgyi, 1929) (Wilson et al., 1993; Belardinelli et al., 1995), slow atrioventricular conduction (negative dromotropism) (Smith, 1989; Pitkanen et al., 1992; Wagner et al., 1998; Davies, 1999; Canyon and Dobson, 2004; David et al., 2007), and they each possess coronary vasodilatory (Berne, 1963; Schrader, 1990; Ely and Berne, 1992; Travis et al., 1999), anti-ischemic (Tosaki et al., 1988; Lasley et al., 1990; Downey et al., 1993; Kamiyama et al., 1995; Jovanovic et al., 1997, 1998; McCully et al., 1998; Vinten-Johansen et al., 1999, 2005; Kanchi et al., 2004; Hove et al., 2007; Wang et al., 2007), anti-arrhythmic (Camm and Garratt, 1991; Alexander et al., 1999; Kucera et al., 2002; Wyman et al., 2004; Jacobson and Gao, 2006) and anti-inflammatory properties (Goldstein et al., 1977; McGregor et al., 1980; Tomoda et al., 1990; Sasagawa, 1991; Cronstein et al., 1992; Das and Misra, 1992a,b; Mikawa et al., 1997; Vitola et al., 1997; Hollmann and Durieux, 2000; Hollmann et al., 2001; Huang et al., 2002; De Kalver et al., 2003; Vinten-Johansen et al., 2003; Halkos et al., 2004; Hasko and Cronstein, 2004; Hollmann et al., 2004; Lenfant et al., 2004; Lappas et al., 2005; Amir et al., 2006; Cassuto et al., 2006; Takahashi et al., 2007; Hasko et al., 2008) and possible coagulation correcting effects (Cooke et al., 1977; Borg and Modig, 1985; Stewart, 1993; Deguchi et al., 1998; Tobias et al., 1999; Hollmann and Durieux, 2000; Broussas et al., 2002; Pinet et al., 2002; Evans et al., 2003; Lappas et al., 2005; Sands and Palmer, 2005; Kreckler et al., 2006) (see Figure 7). Possible cross-talk between A and L may also occur as was recently shown by Benkwitz et al. (2003). Since the adenosine receptors and Na^+^ fast channels are common among most tissues of the body, the clinical applicability of the AL (and Mg^2+^) concept may confer multi-organ protection such as brain, spinal cord, kidney, liver, GI tract, muscle, and lung (Figure 7).

### Advantages of polarized vs. depolarized arrest and reanimation

“Thus maneuvers that precluded activation of the Na^+^ channels, for example, holding the resting membrane potential at −80 mV significantly increased time to cell death or prevented contracture entirely.”

Ward et al. (2006)

The advantage of the polarized arrest and reanimation concept arises from fewer membrane channels, pores and exchangers being open or activated compared with the depolarized state (Chambers and Hearse, 2001; Dobson and Jones, 2004). Depolarised cells are electrically more unstable because many voltage-dependent membrane channels (Ca^2+^, Na^+^ window current, K^+^), pores and exchangers (Na^+^/H^+^ and Na^+^/Ca^2+^) are activated (Kondo, 1987; Carmeliet, 1999; Baczko et al., 2003), which can lead to Na^+^ and Ca^2+^ loading, endothelial activation, coronary artery constriction, arrhythmias and stunning. As mentioned earlier, high K^+^-induced membrane depolarization can lead to the superoxide production in endothelial and vascular smooth muscle cells, platelet aggregation, macrophage and neutrophil activation (Sohn et al., 2000), and underlying tissue damage (Al-Mehdi et al., 1997). In 2006, Ward and colleagues (Ward et al., 2006) reported that emigrated neutrophils, after adhering to ventricular myocytes, caused an immediate membrane depolarization, which contributed to an inflammatory-linked generation of arrhythmias, tissue injury and contractile dysfunction (Ward et al., 2006). Ward et al., further showed that holding the cell membrane potential around the natural “resting” polarized state reduced damage from inflammatory attack (Ward et al., 2006). Thus maintaining a “polarized” membrane potential, or at least stabilizing the voltage during times of stress like global and regional ischemia, appears to be a key factor in preventing the endothelium from activating and triggering a local inflammatory response with increased levels of pro-inflammatory cytokines (IL-1, IL-6, and TNF-α) and other immunologic and coagulopathic derangements.

### Placing the heart in a “natural” state of therapeutic suspended animation

#### AL cardioplegia: pre-clinical data

Our early preclinical data using the isolated rat heart (Dobson and Jones, 2004) and in the canine model of cardiopulmonary bypass (Corvera et al., 2005) supported the AL concept as a new cardioplegic formulation in warm and cold crystalloid and blood environments. In 2004, Dobson and Jones showed that AL cardioplegia, “clamped” the membrane potential close to its resting voltage of −83 mV, as was predicted on theoretical grounds. They also showed that AL cardioplegia arrested the heart for up to 4 h at 29°C with small 30% increases in coronary vascular resistance (CVR), 85–100% recovery of heart rate and systolic pressure, and 70–80% recovery of cardiac output, while only 17% of hearts arrested with St. Thomas' Hospital solution No 2 (Plegisol®) survived (Dobson and Jones, 2004). Proof-of-concept was further demonstrated using all-blood AL cardioplegia in the canine model of cardiopulmonary bypass (Corvera et al., 2005), and in 2007, we reported that AL cardioplegia can be delivered continuously or intermittently at 33°C with no significant loss in functional parameters (Sloots et al., 2007).

**Figure 8:**
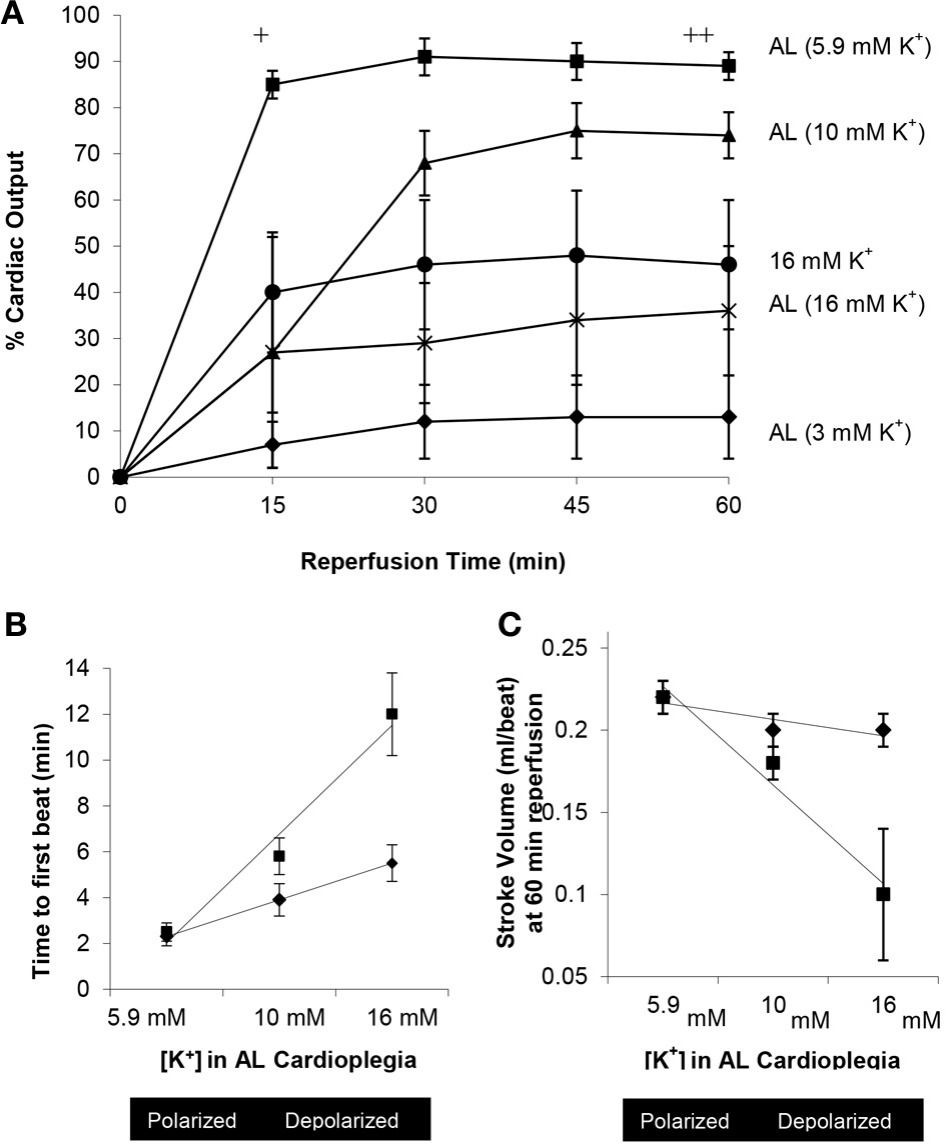
**Effect of increasing extracellular potassium in AL cardioplegia on return of cardiac output, time to first beat and stroke volume after 1 and 2 h arrest at 32–33°C in the isolated working rat heart [Adapted from Sloots and Dobson (2010)]. (A)** Percentage recovery of cardiac output in following 2 h arrest at 32–33°C. ^+^AL (5.9 mM K^+^) *p* < 0.01 (One-Way analysis of variance) compared with AL (3, 10, 16 mM K^+^) and 16 mM K^+^ alone. ^++^AL (5.9 mM K^+^) *p* < 0.01 (One-Way analysis of variance) compared with AL (3 mM K^+^); *p* < 0.05 (repeated measures) compared with AL (3,16 mM K^+^) and 16 mM K^+^ alone. ^*^AL (10 mM K^+^) *p* < 0.01 (repeated measures) compared with AL (3 mM K^+^). **(B)** Relationship between the time to first beat at reanimation after 1 or 2 h arrest and the concentration of potassium in AL cardioplegia solution (5.9, 10, and 16 mM K^+^). **(C)** Relationship between stroke volume and increasing concentrations of potassium in AL cardioplegia solution (5.9, 10, and 16 mM K^+^) at 60 min reperfusion after 1 and 2 h arrest.

In 2010 Sloots and Dobson (Sloots and Dobson, 2010) examined the effect of varying levels of extracellular potassium in AL cardioplegia (0.1, 3.0, 5.9, 10, 16 mM K^+^) on the membrane potential (φ), CVR, incidence of arrhythmias, heart rate, systolic pressures, aortic and coronary flows, cardiac output, time to first beat, and stroke volume after 1 and 2 h of arrest at 32–33°C (Figures 8A–C). Sloots and Dobson showed that warm AL cardioplegia is most efficient under *normokalemic* conditions when the myocardial cell membrane potential is close to its resting state. Hearts arrested using higher K^+^ levels (depolarizing cardioplegia) or interestingly, using lower K^+^ levels (hyperpolarizing cardioplegia), had significantly higher CVR's, experienced reanimation arrhythmias and were “slow-to-recover” with significant losses in left ventricular (LV) function, SV and contractility (Sloots and Dobson, 2010). *LV functional loss was highly correlated with high or very low potassium levels in the cardioplegia* (Figures 8A–C). Nearly 40% of AL (0.1 mM K^+^) and 25 mM K^+^ alone hearts failed to return HR, developed pressures or CO after 1 h arrest at 32–33°C. In 2009, Rudd and Dobson advanced the AL cardioplegic concept as a new paradigm for orthotopic heart transplantation, and showed that arrest and reanimation was versatile at both cold (4°C) and warmer (28–30°C) temperatures compared with Celsior solution (Rudd and Dobson, 2009). In 2011, Rudd and Dobson further showed that the rat heart could be placed in suspended animation with AL, insulin and melatonin for 8 h in cold static storage (4°C) and returned 80% of left ventricular function compared to two FDA approved preservation solutions, Custodiol-HTK and Celsior, that could barely return 10% left ventricular function (Rudd and Dobson, 2011b). Further studies are underway to further improve ALM™ cardioplegia and preservation solutions.

#### Reanimation with ALM: key to restoring a broken heart

The AL concept of voltage control and defending ionic and metabolic balance may also be applicable for reperfusion following heart surgery or organ storage. Canyon and Dobson showed that pre-treatment (i.e., preconditioning) with intravenous low dose AL infusion 5 min prior and during 30 min of regional ischemia led to superior protection from acute ischemic damage resulting in no death, no severe arrhythmias and a significant 38% decrease in infarct size (Canyon and Dobson, 2004). In contrast, fifty-eight percent (58%) of saline controls died from ventricular fibrillation (Canyon and Dobson, 2004). In another study, the adenosine A_1_ agonist 2-chloro-N6-cyclopentyladenosine (CCPA) was substituted for adenosine, and the CCPA-lidocaine combination showed remarkable infarct size reduction of over 80%, which was equivalent to that of ischemic preconditioning itself (Canyon and Dobson, 2005). In 2006, Canyon and Dobson showed using ^31^P nuclear magnetic resonance (NMR) that the protective effects of AL constant infusion therapy were expressed as preservation of high-energy phosphates (ATP, PCr) in the area-at-risk of the left ventricle, and this was explained by a down-regulation of the myocardium during ischemia (Canyon and Dobson, 2006). This *in vivo* study is consistent with the energy-sparing properties shown in the cardioplegia studies. That the ATP and PCr concentrations were maintained during ischemia supports our hypothesis that AL pretreatment/ischemia therapy may provide protection by ensuring better metabolic and electrical matching in different heterogeneous regions of the heart, and may be useful for off-pump surgery or angioplasty.

In 2011 Rudd and Dobson combined the polarizing AL arrest and reperfusion strategy to cold preservation of the heart. Rewarming the donor rat heart after 6 or 8 h cold static storage for 5 min *with a normokalemic, oxygenated, polarizing AL arrest solution* resulted in significantly higher aortic flow, coronary flow, and cardiac output compared with controls and other FDA-approved solutions (Rudd and Dobson, 2011a,b). Interestingly, rewarming cold hyperkalemic Celsior hearts with polarizing AL solution *reduced stunning* compared with the hyperkalemic “hot shot” (Rudd and Dobson, 2011a). With this strategy Rudd and Dobson added a fifth new approach to minimize myocardial damage *during rewarming and implantation* of donor hearts, and possibly other organs. These five strategies are (1) temperature control, (2) hemodynamic control (unloaded nonworking mode), (3) pressure control, (4) reperfusate control, and (5) membrane voltage control using normokalemic, polarizing strategies (Rudd and Dobson, 2011a).

## ALM™ cardioplegia: from concept to clinical trials

### The verona trial: first prospective, randomized trial

The Verona trial was an important trial as it was the first randomized prospective clinical trial using ALM™ (with insulin) in microplegia compared to 4:1 Buckberg solution (Onorati et al., 2012). Eighty adult patients were selected from a total of 878 and were emergency cases with unstable angina and required CABG surgery only (not valve). Patients were randomized to receive either standard 4:1 blood (“Buckberg”) cardioplegia or all-blood cardioplegia (microplegia) enhanced with ALM and insulin (Onorati et al., 2012). The authors found that ALM-insulin significantly (1) reduced transmyocardial troponin-I and lactate release, (2) improved post-operative cardiac function, (3) increased the incidence of return of spontaneous rhythm at declamping, (4) reduced the use of higher doses of inotropic agents, (5) reduced blood transfusions and use of most blood products, and (6) decreased the length of both ICU and total hospital stay compared to the 4:1 Buckberg blood cardioplegia formulation (Table 1). There was no mortality in either group, and therefore mortality is not the only important end point with impact. The authors note that further multi-center trials are required, and it may be of interest to include a full cost saving analysis since in their study the one full day less in the ICU in the USA would represent a savings of $4000–10,000 per patient (Rapoport et al., 2003), along with the 50% reduction in use of blood products with direct and indirect costs ranging between $522 and $1183 per unit of red cells (Shander et al., 2010). From a safety perspective, blood transfusions are not “outcome neutral” and may negatively impact on patient recovery in the ICU (Gong et al., 2005).

**Table 1:** **Prospective Randomized Human trial data comparing Microplegia ALM(I) with 4:1 Buckberg Solution**.

	**ALM(I) Microplegia (*n* = 40)**	**Buckberg (4:1) (*n* = 40)**	***P*-level**	**Significance**
**MYOCARDIAL PROTECTION**				
• Troponin (during operation, 6, 12, and 48 h	0.5–2.5	1.25–7.0	0.001	✓
• Lactate (same as above)	0.7–1.5	1.5–2.5	0.0001	✓
**CARDIAC PERFORMANCE**				
• Cardiac Index (cardiac output/m^2^)	3.9	2.75	0.0001	✓
• Systolic Function (cardiac efficiency (CCE), pressure wave profile (Δp/Δt), wall motion score index (WMSI)	↑CCE ↑Δ p/Δ t ↑WMSI	Lower Systolic Function	<0.05	✓
• Diastolic Function (E-wave, E/A,and Ea at TD)	↓E-wave ↓Ea velocity ↑E, ↑E/A and ↑Ea	Lower Diastolic Function	<0.05	✓
• Return Spontaneous Rhythm at Declamping (%)	78	28	0.001	✓
• Perioperative acute MI (%)	2.5	5.0	1.0	NS
• Low output syndrome (%)	5	12.5	0.432	NS
• Balloon support (IABP)(%)	5	10	0.675	NS
• Use of Inotropes (%)	57%	85%		✓
• Length of time inotropes (h)	31	51	0.041	✓
**BLOOD TRANSFUSION**				
• % patients needing blood (pts)	25	58	0.001	✓
• Red Packed cells (U/pt)	0.3	1.3	0.001	✓
• Fresh Frozen plasma	0.2	1.3	0.001	✓
• Platelets (Units/pt)	0.02	1.3	0.149	NS
**ICU AND HOSPITAL STAY**				
• ICU (H)	48	73	0.015	✓
• Total hospital (days)	7.4	9.4	0.002	✓
• Mortality	No Mortality	No Mortality		NS

*Data are post-operative with significance level on RHS column (Onorati et al., 2012). The Italian authors confusingly refer to trend when talking about significant differences (P < 0.05). Actual p-values are included above. ALM(I) is adenosine, lidocaine and Mg^2+^, and Insulin*.

### ALM™ protection in a 4× redo patient requiring 7 h cardioplegic arrest

ALM cardioplegia was used in a high risk case at Intermountain Medical Center, Salt Lake City (O'Rullian et al., 2008). The patient was a 71 year-old four-time redo male who was diagnosed with prosthetic valve endocarditis of both aortic and mitral valves, and subsequently required a re-operative aortic and mitral valve replacement. The patient was placed CPB and the heart was arrested within 15 s using normothermic all-blood AL microplegia (AL with 25 mEq/L KCl, 16 mM MgSO4). Arrest was maintained with 7°C near-continuous retrograde AL microplegia using 0–2 mEq/L added potassium. After four cross-clamp periods from bleeding and valve seating complications, the heart was reanimated with a normokalemic blood “hot-shot” comprised of two liters of AL with magnesium but without KCl. Ultimately, hemostasis was achieved and the patient was weaned from CPB without requiring an intra-aortic balloon pump, on a standard regimen of inotropic agents, and was extubated after 12 h. The patient was on CBP for 9 h and 50 min, with a total cross-clamp time of 7 h. The heart was perfused with a total of 72 liters all-blood AL microplegia. However, the total crystalloid volume used for the additives and potassium was 250 ml. Post-operatively, there was no systemic hyperkalemia (5.1 mmol/L) no hemodilution (hematocrit 24%), and no remarkable edema. This single case report shows how using AL at physiological potassium levels may find utility in very high-risk patients with prolonged bypass and cardioplegia volumes. Curiously, adenosine is known to have a short-half life in blood *in vitro* (reported to range from 4 to 10 seconds), however, this does not appear to limits AL's ability to arrest or protect the heart in the pediatric or high-risk case study indicating protection in animal and human studies may come from continuous activation of the adenosine receptors together with voltage-dependent Na+ fast channel modulation.

### Safety of AL crystalloid cardioplegia for pediatric cardiac surgery

Cardioprotection during pediatric surgery represents a major unmet need. Jin and colleagues (Jin et al., 2008) conducted a prospective, single-center, randomized clinical trial involving 134 pediatric patients with low-risk congenital heart disease. On the day of surgery, surgeons and intensive care physicians were blinded to three crystalloid cardioplegic formulations; high potassium group (K^+^, 20 mM), high potassium AL group (20 mM K^+^, 0.7 mM A, 0.7 mM L) and moderate potassium AL group (10 mM K^+^, 0.7 mM A, 0.7 mM L). Around 80% of patients had ventricular septal defects (VSD) and 60% had concomitant pulmonary hypertension. There were no significant differences among the three groups in defect types, age, sex or body weight or hemodynamics before CPB. The arrest protocol comprised a single hypothermic (ice-cold) antegrade infusion of 20 ml kg^−1^ crystalloid cardioplegia administered over a 2 min period (100 ml per min for a total volume of ~200 ml). The cardioplegia was aspirated by means of vigorous suction at the coronary sinus to prevent distribution into the systemic circulation.

There were no significant group differences in time to arrest, cross-clamp time, cardiopulmonary bypass (CPB) time, peri-operative hematocrit, fluid output at the end of the operation, mechanical ventilation time, total mediastinal drainage, ICU time (~2.5 days) and post-operative hospital time (11–13 days). However, there was a trend toward higher number days spent in hospital and high potassium in the cardioplegia. The total post-operative hospital time for the high K^+^ group was 12.7 ± 1.2 days, followed by high K^+^(AL) (11.8 ± 0.6 days) and the lowest was in the moderate 10 mM K^+^(AL) group (11.1 ± 1.4 days) (Jin et al., 2008). Furthermore, there were significantly higher systolic and pulse pressures following CPB in the 10 mM K^+^(AL) group compared with the non-AL 20 mM K^+^ group, and again after modified ultrafiltration. Similarly, serum Troponin I levels were significantly lower in the moderate 10 mM K^+^(AL) group compared to both the non-AL 20 mM K^+^ group and High K^+^(AL) group in the first 12 h following cross-clamp removal. The 10 mM K^+^(AL) group had serum troponin levels that were 37, 48, 63, and 62 of the values measured in the non-AL K^+^ group at 1, 3, 6, and 12 h respectively. Jin et al., also reported a 30% lower use of post-operative inotropes in the 10 mM K^+^(AL) group compared to non-AL 20 mM K^+^ group (Jin et al., 2008). Unfortunately, Jin et al. (2008) did not test AL with physiological potassium concentrations (~5 mM K^+^). The authors concluded that AL in hyperkalemic and moderately hyperkalemic crystalloid cardioplegia was safe and effective, and that 10 mM K^+^(AL) cardioplegia formulation was associated with improved protective effects in pediatric patients.

## Beyond cardioplegia: ALM ™ as a biological response modifier in trauma, inflammation, coagulopathy, and sepsis

Since the heart returns such vigorous activity with less need for cardioversion and inotropic support following heart surgery, at James Cook University we began to investigate the ability of non-arresting concentrations of ALM to rescue and stabilize the heart following severe blood loss and shock. The key point is that ALM at high concentrations “arrests” the heart, *and at lower concentrations resuscitates the heart*. In our first hemorrhagic shock study, we confirmed that colloids 6% and 10% hetastarch or 6% dextran were problematic on cardiac stability, hemodynamics and all led to increased mortality in the rat model (Letson and Dobson, 2011a,b). In a second study, we showed that ~1 ml/kg bolus (~8 drops per 300 g rat) of 7.5% NaCl/ALM (without colloids) solved this problem by rescuing the heart and resuscitating MAP into a hypotensive range (Letson and Dobson, 2011a,b), and further it fully corrected acute traumatic hypocoagulopathy (Letson et al., 2012b). In a third study, we reported an unexpected 100% survival in the 7.5% NaCl ALM group after 60% blood loss with higher MAP and few or no arrhythmias compared with 7.5% NaCl alone, or any other treatment groups (Letson and Dobson, 2011c). ALM also has the advantage of displaying potent anti-inflammatory properties by reducing the priming and activation of neutrophils (Shi et al., 2012).

In 2012, Letson and colleagues successfully translated ALM ™ resuscitation from rat into the pig model of 75% blood loss (Letson et al., 2012a). Small volume 4 ml/kg 7.5% NaCl ALM resuscitated MAP into the hypotensive region with a 2-fold increase in stroke volume, a 34% fall in blood lactate and a 43% higher O_2_ delivery. After the shed blood (~2L) was returned whole body O_2_ consumption fell, systemic vascular resistance increased 30%, and urine output in the ALM group increased threefold compared with 7.5% NaCl treatment (Letson et al., 2012a). Importantly, small volume 7.5% NaCl (vehicle control) was not optimal in rat or pig, which is consistent with the recent randomized, multi-center trial that reported no significant benefit of 250 ml 7.5% NaCl, or 7.5% NaCl 6% Dextran-70 compared to normal saline for early resuscitation of hemorrhagic shock (Bulger, 2011). The cardiac rescue potential of small volume 7.5% NaCl ALM was further demonstrated by Grandfeldt and colleagues who showed that a 20 ml bolus of 7.5% NaCl ALM (0.5 ml/kg) significantly reduced fluid requirement by 40% to reach a target MAP of 50 mmHg in pig model following 75% blood loss (Granfeldt et al., 2012), and a 10 ml bolus injection of 0.9% NaCl AL (no Mg^2+^) with return of shed blood led to a significant 27% drop in whole body O_2_ consumption and improved cardiac and renal function (Granfeldt et al., 2012). Lastly, pilot rat and sepsis studies show that an IV infusion of ALM ™ improves cardiac function, reduces neutrophil activation and TnF alpha, and reduces whole body O_2_ consumption. Further work is required to test if these strategies are safe and efficacious in the preshospital civilian environment or on the battlefield where few saline based or colloid-based resuscitation fluids drugs currently work (Blackbourne et al., 2010). However, since cardiac surgery involves trauma, inflammation, and coagulopathy, it is possible that the ALM ™ polarized concept may be therapeutic and provide whole body protection for the patient as was indicated from aspects of the Verona trial. A future goal will be to examine the effect of ALM ™ infusion to protect the brain during circulatory arrest for aortic reconstruction surgery in pediatrics and adults.

## Concluding remarks

Despite groundbreaking advances in global health care, cardiac surgeons, anesthetists and perfusionists are confronted with a new older and sicker patient with an increasing risk profile. The new patient may require complex redo surgery or other emergency procedures from failed angioplasties, cardiogenic shock, or congenital defects. Depolarizing potassium cardioplegia may not provide optimal perioperative protection for today's adult or pediatric patient. New innovations are required and the ALM ™ concept with other additions may offer a new normokalemic polarizing strategy for heart and whole body protection. While the outcomes from early human trials appear promising, further prospective, randomized multi-center clinical trials are required.

### Conflict of interest statement

Geoffrey P. Dobson is the inventor of AL and ALM platform, and ALM has been trademarked. The other authors declare that the research was conducted in the absence of any commercial or financial relationships that could be construed as a potential conflict of interest.
